# Potential of Mitochondrial Ribosomal Genes as Cancer Biomarkers Demonstrated by Bioinformatics Results

**DOI:** 10.3389/fonc.2022.835549

**Published:** 2022-05-26

**Authors:** Shunchao Bao, Xinyu Wang, Mo Li, Zhao Gao, Dongdong Zheng, Dihan Shen, Linlin Liu

**Affiliations:** ^1^ Department of Radiotherapy, Second Hospital of Jilin University, Changchun, China; ^2^ Department of Breast Surgery, Second Hospital of Jilin University, Changchun, China; ^3^ Nuclear Medicine Department, Second Hospital of Jilin University, Changchun, China; ^4^ Department of Cardiovascular Surgery, Second Hospital of Jilin University, Changchun, China; ^5^ Medical Research Center, Second Hospital of Jilin University, Changchun, China

**Keywords:** mitochondrial ribosome, bioinformatics, cancer, biomarker, apoptosis, energy metabolism

## Abstract

Next-generation sequencing and bioinformatics analyses have clearly revealed the roles of mitochondrial ribosomal genes in cancer development. Mitochondrial ribosomes are composed of three RNA components encoded by mitochondrial DNA and 82 specific protein components encoded by nuclear DNA. They synthesize mitochondrial inner membrane oxidative phosphorylation (OXPHOS)-related proteins and participate in various biological activities *via* the regulation of energy metabolism and apoptosis. Mitochondrial ribosomal genes are strongly associated with clinical features such as prognosis and foci metastasis in patients with cancer. Accordingly, mitochondrial ribosomes have become an important focus of cancer research. We review recent advances in bioinformatics research that have explored the link between mitochondrial ribosomes and cancer, with a focus on the potential of mitochondrial ribosomal genes as biomarkers in cancer.

## 1 Introduction

In human cells, the vast majority of mitochondria-associated proteins are encoded by nuclear genes and synthesized by cytoplasmic ribosomes. Mitochondria, as endosymbiotic organelles, retain the characteristics of the original genomic DNA of their bacterial ancestors and possess relatively independent gene expression mechanisms (1). Human mitochondrial DNA (mtDNA) encodes two ribosomal RNAs (rRNAs) and a group of transfer RNAs (tRNAs) as well as oxidative phosphorylation (OXPHOS)-related proteins. These mitochondrially encoded proteins are located within the inner mitochondrial membrane and are synthesized by mitochondrial ribosomes (2). The mitochondrial ribosome differs from bacterial and cytoplasmic mitochondria with respect to complexity, structure, and mechanisms of action. It is composed of three RNA components encoded by mitochondrial DNA and 82 specific protein components encoded by nuclear DNA (3, 4). Over the past decade, the roles of mitochondrial ribosomal proteins in apoptosis and cellular energetics have been revealed (5, 6). Importantly, the inhibition of cell death, proliferative signaling, and deregulation of cellular energetics are hallmarks of cancer (7).

Margaret Dayhoff (1925–1983), an American physical chemist known as “the father of bioinformatics”, was the first to apply computational methods to the biological realm (8). Relying on advances in both molecular biology and computer science, bioinformatics has been evolving over several decades. The application of microarray technology, next-generation sequencing, and big data analysis techniques has revealed the important roles of mitochondrial ribosomal proteins in cancer biology.

This review examines the link between mitochondrial ribosomes and cancer. In particular, the composition, structure, and function of the mitochondrial ribosome are described. Bioinformatics tools, including relevant databases and methods for sequencing and analysis, are described with a focus on their application to the mining of candidate mitochondrial ribosomal protein biomarkers for malignancy. This review adds a bioinformatics perspective to research on mitochondrial ribosomes in relation to malignancies.

## 2 Mitochondrial Ribosomes

### 2.1 Components and Structure

**Figure 1:**
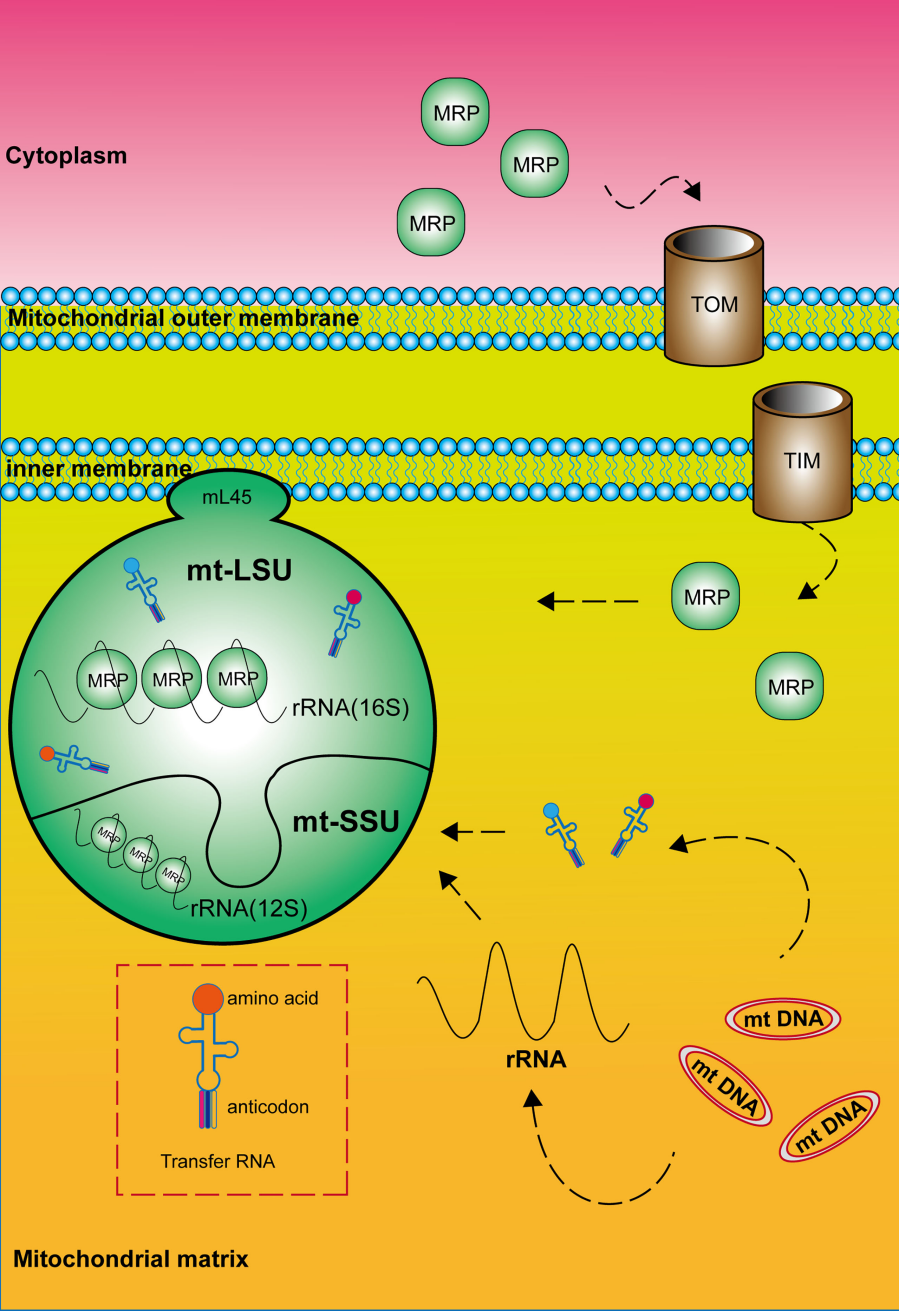
Composition and structure of the mitochondrial ribosome. The mature mitochondrial ribosome, which consists of a large and a small subunit, is permanently anchored to the inner mitochondrial membrane by *MRPL45* (mL45) of the large subunit. Mitochondrial ribosomal proteins are synthesized within the cytosol and enter mitochondria mediated by the transmembrane transport complex including Translocase of the Outer mitochondrial Membrane (TOM) and Translocase of the Inner Membrane (TIM). RNA components are encoded by mitochondrial DNA.

In all organisms, the mature mitochondrial ribosome is composed of a large subunit (LSU) responsible for catalyzing peptidyl transferase reactions and a small subunit (SSU) providing a platform for mRNA binding and decoding (**Figure 1**). The 28S mt-SSU of the mammalian mitochondrial ribosome consists of one 12S rRNA and 29 mitochondrial ribosomal proteins (MRPs), whereas the 39S mt-LSU consists of one 16S rRNA, 48 MRPs, and one structural tRNA (9, 10). Using deep RNA sequencing, Alan Brown identified this structural tRNA as mt-tRNA ^Val^, a molecule whose position resembles that occupied by 5S rRNA in the cytoplasmic ribosome (11). In addition, 16 mt-SSU proteins and 28 mt-LSU proteins are homologous to their counterparts in *Escherichia coli*, and the rest are mitochondria-specific proteins (12, 13). These nuclear-encoded subunit proteins are synthesized within the cytosol and enter mitochondria for subsequent assembly, mediated by chaperones and the transmembrane transport complex including Translocase of the Outer mitochondrial Membrane (TOM) and Translocase of the Inner Membrane (TIM) (14, 15). Unlike cytoplasmic ribosomes, the majority of mitochondrial ribosomes are permanently anchored to the inner mitochondrial membrane by mL45 (*MRPL45*) of the large subunit (4). The large and small subunits are connected by three protein–protein and six protein–RNA inter-subunit bridges, whereas bridges in bacterial and eukaryotic cytoplasmic ribosomes mainly consist of conserved RNA–RNA interactions (16). In addition, mitochondrial ribosomes differ from cytoplasmic and bacterial ribosomes by a high RNA-to-protein ratio (17). Finally, two relevant publications are cited that provide the nomenclature of mitochondrial ribosomal proteins for readers to consult (18, 19).

### 2.2 MRPs and Energy Metabolism

**Figure 2:**
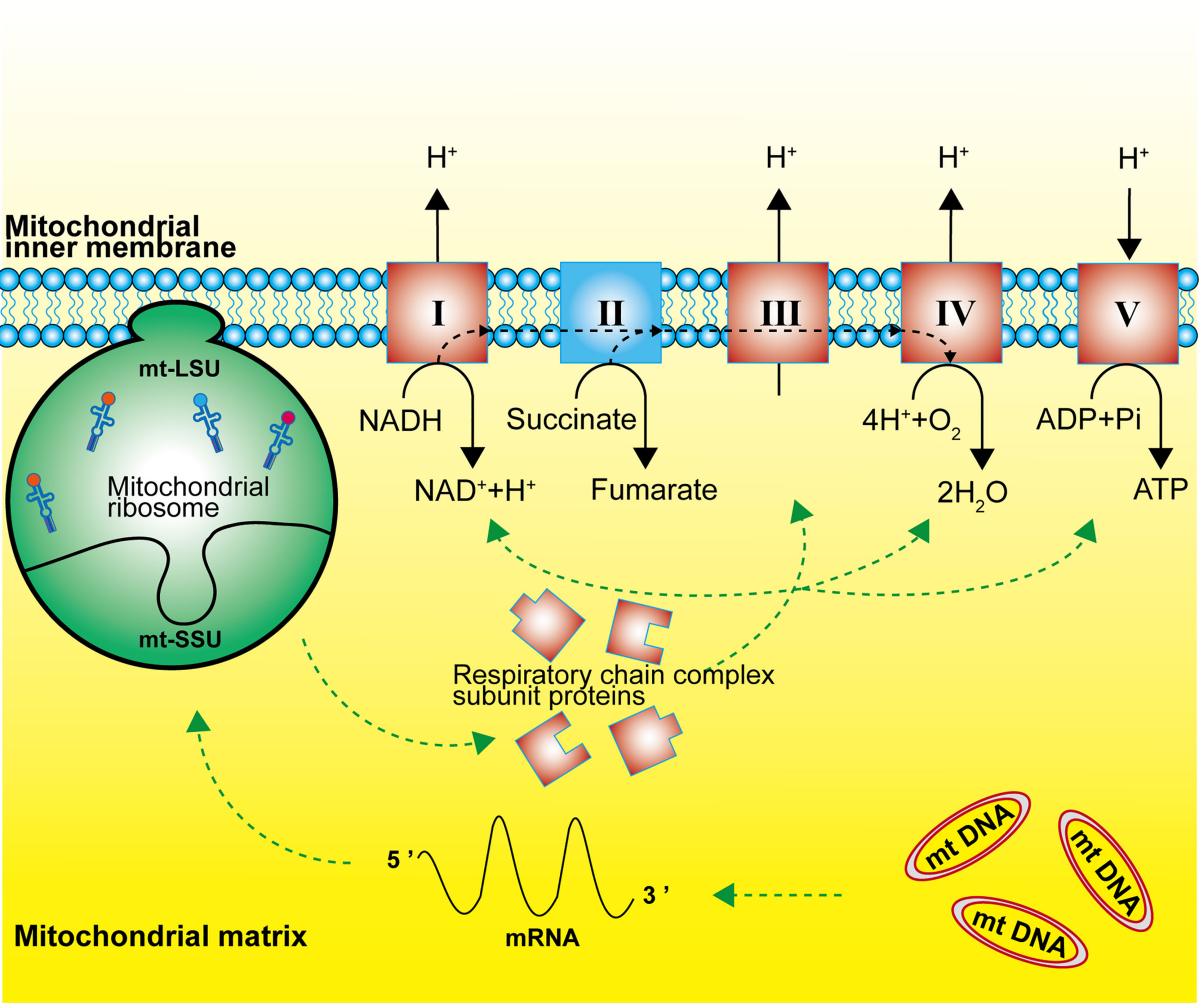
Function of the mitochondrial ribosome. Mitochondrial ribosomes synthesize the subunit proteins of OXPHOS complex I, III, IV and V, which are encoded by the mitochondrial DNA. The dashed black line represents the flow of electrons.

Mitochondrial ribosomes are responsible for the translation of 13 subunit proteins of OXPHOS complex I, III, IV and V, which are encoded by the mitochondrial DNA. These proteins located in the inner mitochondrial membrane include seven subunits of complex I (NADH: ubiquinone oxidoreductase), one subunit of complex III (ubiquinone: cytochrome c oxidoreductase), three subunits of complex IV (cytochrome C: oxidoreductase), and two subunits of complex V (ATP synthase) (**Figure 2**) (2, 11, 20). Single-stranded mRNAs formed after mtDNA transcription are enriched at the SSU neck, where the pentatricopeptide repeat protein *MRPS39* (mS39) recognizes and binds to the 5′ end of the mRNA and activates the translation machinery (21, 22).

In mammalian cells under normal conditions, more than 80% of cellular ATP requirements are met by ATP generated by oxidative phosphorylation (OXPHOS). The OXPHOS metabolic pathway generates ATP by transporting electrons along a series of transmembrane protein complexes in the inner mitochondrial membrane, called the electron transport chain (ETC). Energy transport is achieved by the flow of electrons between complex I, complex II, coenzyme Q10, complex III, cytochrome c, and complex IV, with oxygen as the terminal electron acceptor (23). A methylation study has revealed a unique mechanism by which arginine and lysine methylation of *MRPS23* promote breast cancer metastasis by regulating OXPHOS (24). Bioinformatics analyses have revealed that *MRPS5* is closely related to the function of OXPHOS complex I and the acetylation status of *MRPS5* is directly regulated by the NAD^+^-dependent deacetylase sirtuin-1 (*SIRT1*), indicating that the *SIRT1/MRPS5* axis is involved in metabolic reprogramming and tumor progression (25). *MRPL13* expression is reduced in OXPHOS-deficient hepatoma cells (SNU354 and SNU423 cell lines), and the specific inhibition of mitochondrial glycosomal translation by siRNA-mediated knockdown of *MRPL13* decreases expression of the OXPHOS complex IV subunit protein *COX2 *(26).

Metabolic reprogramming in cancer cells confers the ability to adjust metabolic pathways to support heterogeneous biological processes. According to the Otto Warburg theory, glycolysis is upregulated in cancer cells compared to that in normal cells (7). However, this does not mean that oxidative phosphorylation is universally downregulated in cancer. A meta-analysis has suggested that the average contribution of OXPHOS to ATP production is 80% in normal cells and 83% in cancer cells (27), consistent with *in vivo* data from a study by Vaupel et al., who showed that the mitochondrial respiratory capacity is not always functionally impaired (28). Variation in the contribution of OXPHOS among cancer types may be explained by differences in the mtDNA content. In particular, the mtDNA content is higher in many cancer tissues than in normal tissues, including tissues from patients with endometrial cancer, colorectal cancer, ovarian cancer, prostate cancer, head and neck squamous cell carcinoma, lung adenocarcinoma, esophageal squamous cell carcinoma, and thyroid cancer (20, 29). There is growing evidence that some cancers are critically dependent on OXPHOS, and inhibiting OXPHOS can effectively target specific cancer subtypes. For example, patients with diabetes receiving the anti-diabetic drug metformin have a lower incidence of cancer and a better prognosis than patients not receiving metformin (30, 31). *In vitro* studies suggest the effect of metformin is mediated by the inhibition of OXPHOS complex I, resulting in decreased ATP production by cancer cells (32).

Since the expression analyses of genes encoding OXPHOS related proteins in mtDNA may not fully reflect the function of OXPHOS, proteomic or metabolomic approaches are required to fully characterize OXPHOS activity. Furthermore, given that mitochondrial ribosomes are responsible for the synthesis of OXPHOS related proteins, relevant studies targeting the expression of genes encoding mitochondrial ribosomal proteins and the function of MRPs should not be overlooked.

### 2.3 MRPs and Apoptosis

One of the hallmarks of tumorigenesis and tumor progression is the evasion of apoptosis in cancer cells, and traditional chemoradiotherapy largely relies on the induction of cancer cell apoptosis. The mitochondrial pathway is an important apoptosis pathway and is regulated by pro-apoptotic factors (such as Bax and Bak) as opposed to anti-apoptotic factors (such as Bcl-2 and Bcl-xL) from the Bcl-2 (B-cell Lymphoma 2) protein family (33, 34). Subsequent mitochondrial outer membrane permeabilization (MOMP) and the release of mitochondrial pro-apoptotic proteins (cytochrome c, SMAC, and OMI) into the cytoplasm results in the activation of the caspase family of proteases, which promote apoptosis *via* a cascade of reactions, leading to cell death (35, 36). Among the mitochondrial ribosomal proteins, *MRPL41* (mL41), *MRPS29* (mS29), and *MRPS30* (mS30) have been implicated in the regulation of apoptosis.

**Figure 3:**
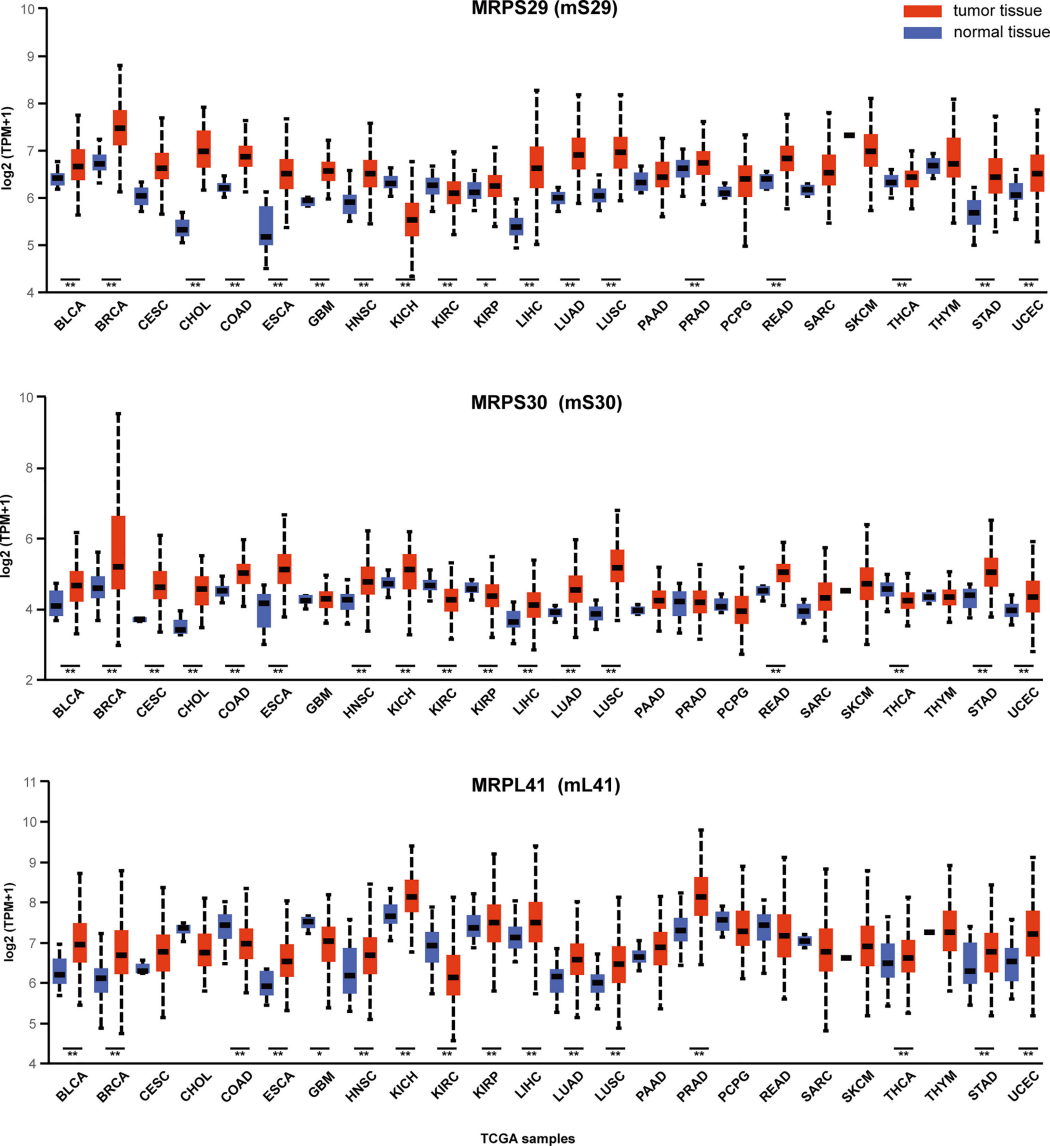
Expression of MRPS29, MRPS30, MRPL41 across TCGA cancers. Three representative mitochondrial ribosomal genes are differentially expressed in cancer. TPM is the normalization of gene reads derived from high-throughput sequencing (39). (*p < 0.05; **p < 0.01).TPM, Transcripts Per Kilobase Million; BLCA, Bladder urothelial carcinoma; BRCA, Breast invasive carcinoma; CESC, Cervical squamous cell carcinoma; CHOL, Cholangiocarcinoma; COAD, Colon adenocarcinoma; ESCA, Esophageal carcinoma; GBM, Glioblastoma multiforme; HNSC, Head and Neck squamous cell carcinoma; KICH, Kidney Chromophobe; KIRC, Kidney renal clear cell carcinoma; KIRP, Kidney renal papillary cell carcinoma; LIHC, Liver hepatocellular carcinoma; LUAD, Lung adenocarcinoma; LUSC, Lung squamous cell carcinoma; PAAD, Pancreatic adenocarcinoma; PRAD, Prostate adenocarcinoma; PCPG, Pheochromocytoma and Paraganglioma; READ, Rectum adenocarcinoma; SARC, Sarcoma; SKCM, Skin Cutaneous Melanoma; THCA, Thyroid carcinoma; THYM, Thymoma; STAD, Stomach adenocarcinoma; UCEC, Uterine Corpus Endometrial Carcinoma.

In human cells, Bcl-2 blocks the apoptosis-inducing ability of *MRPL41* (mL41) *via* multiple mechanisms, and amino acid residues 13–17 of mL41 are essential for binding to Bcl-2; accordingly, mL41 has also been named BCL-2-interacting mitochondrial ribosomal protein (BMRP). *MRPS29* (mS29), originally also known as death-associated protein 3 (DAP3), is strongly associated with the Fas receptor-associated death-inducing signaling complex. *MRPS30* (mS30) is also known as programmed cell death protein 9 (PDCD9) (37). Although the roles of these three MRPs in the regulation of apoptosis are well-established, studies of their roles in cancer biology are limited. We utilized the visualization tool UALCAN (http://ualcan.path.uab.edu/index.html (38).) to analyze the gene expression data for these three genes from The Cancer Genome Atlas (**Figure 3**). These three representative genes are highly expressed in many malignancies, such as lung and breast cancer (p < 0.01) and can be intensively studied as potential tumor biomarkers. The expression of gene MRPS29 is higher in TCGA gastric adenocarcinoma samples than in controls (normal n = 34; primary tumor n = 415), consistent with the published conclusion of Jia y (40). Interestingly, they are not always highly expressed in cancer. For example, *MRPL41* and *MRPS30* are under expressed in renal clear cell carcinoma (p < 0.01), which may be associated with tumor heterogeneity, but requires further investigation.

## 3 Bioinformatics, Mitochondrial Ribosomes, and Malignancy

In the early 1960s, bioinformatics was developed along with advances in molecular biology and computer science. In the 2000s, major innovations in sequencing technology as well as decreasing costs led to the arrival of the “big data” era, with bioinformatics data mining and management forming a new area of expertise (41). Bioinformatics analytics based on next-generation sequencing aim to apply advanced computational tools and databases to convert sequencing signals into data with interpretable information. The whole analysis process can be simplified into three steps. The first step consists of processing the raw sequencing instrument signal into nucleotide bases and short-read data (42). The second step consists of alignment to the reference sequence or *de novo* assembly of nucleotide reads and subsequent variant detection, etc. (43). The third step relates sample-specific genomic profiles to disparate descriptive annotations, followed by statistical analysis and visualization. A comprehensive review (with classification and description) of major DNA, RNA, and protein related bioinformatics databases with publicly available tools was performed by Chen etc. (44–47).

With respect to bioinformatics and cancer, valuable databases have been established. For example, the Gene Expression Omnibus (GEO http://www.ncbi.nlm.nih.gov/geo/summary/) is a widely used database supported by the National Center for Biotechnology Information (NCBI), which primarily hosts global gene expression data for access by the research community (48). The Cancer Genome Atlas (TCGA http://cancergenome.nih.gov/abouttcga) is the landmark cancer genomics program initiated and funded by the National Institutes of Health to molecularly characterize more than 20,000 primary cancers with the goal of improving cancer diagnosis, treatment, and prognosis prediction (49, 50). The massive genomic, epigenomic, transcriptomic, and proteomic data generated from sequencing are all available for subsequent bioinformatics analyses. TCGA data are based on microarrays (to test nucleic acids and proteins) and next-generation sequencing (for global analyses of nucleic acids), including but not limited to RNA sequencing, DNA sequencing, and reverse-phase protein arrays.

To meet the demand for multi-dimensional comprehensive analyses of massive genomic data, advanced visual analysis methods, such as weighted gene co-expression network analysis (WGCNA), have been developed. New advances in the field of mitochondrial ribosomes and malignancies using common high-throughput sequencing and bioinformatics techniques, coupled with *in vitro* and *in vivo* experimental validation, are described below. We have produced two Tables for readers’ access based on the findings mentioned below (**Tables 1**, **2**).

**Table 1:** Association between mitochondrial ribosomal genes and cancer revealed by high-throughput sequencing.

Sequencing Categories	Molecular Event	*Molecular Signatures*	Cancer	Reference
**RNA sequencing**	Differential Gene Expression	*MRPL13*	Non-small cell lung cancer	(51)
		*MRPS6 MRPS10 MRPS31*	Breast cancer	(52, 53)
		*MRPS23*	Luminal breast cancer	(54, 55)
			Colorectal cancer	(56)
		*MRPL54*	Hepatocellular carcinoma	(57, 58)
		*MRPS12*	Ovarian cancer	(59)
		*MRPS7*	Osteosarcoma	(60)
		*MRPL27*	Lung adenocarcinoma	(61)
		*MRPL9*	Hepatocellular carcinoma	(40)
		*MRPS18A*	Cholangiocarcinoma	(62)
	Gene Fusions	*MRPS31-SUGT1*	Colorectal cancer	(63)
		*MRPS30-ARID2*	Intimal sarcoma	(64)
	LncRNAs	*MRPL23-AS1*	Bladder cancer	(65)
			Hepatocellular carcinoma	(66)
		*LINC00152-USF1/MRPL52*	Oral squamous cell carcinoma	(67)
		*TRIM52-AS1-MRPS18A*	Hepatocellular carcinoma	(68)
**DNA sequencing**	SNPs	*rs2839698*-*MRPL23-AS1* *rs3024270* -*MRPL23-AS1*	Hepatoblastoma	(69)
		*rs4919510-MRPL43*	Colorectal cancer	(70)
	Methylation	*lnc-MRPL3-2*	Neuroblastoma	(71)

**Table 2:** Association between mitochondrial ribosomal genes and clinical features of cancer revealed by bioinformatics analyses.

Clinical Features	Cancer	Molecular Signatures	Reference
**Prognosis**	Head and neck squamous cell carcinoma	*MRPL47*	(72)
	Neuroblastoma	*MRPL11*	(73, 74)
	Non-small cell lung cancer	*MRPL15*	(75)
	Lung adenocarcinoma	*MRPL42*	(76)
	Breast cancer	*MRPL3*	(77)
		*MRPL13*	(78–81)
		*MRPL12* *MRPL13*	(82)
	Gastric cancer	*MRPS5*	(83)
		*MRPL35*	(84)
		*MRPS29*	(85)
		*MRPS17*	(86)
	Hepatocellular carcinoma	*MRPL9*	(87)
		*MRPS12*	(88)
	Cholangiocarcinoma	*MRPL27*	(89)
	Colorectal cancer	*MRPL52*	(90)
		*MRPL35*	(91)
	Ovarian cancer	*MRPL15*	(92)
	Adrenocortical carcinoma	*MRPS23*	(93)
**Metastasis**	Breast cancer	*MRPL52*	(94)
		*MRPL15*	(95)
	Ovarian cancer	*MRPL38*	(96)
**Drug responsiveness**	Muscle invasive bladder cancer	*MRPL4*	(97)

### 3.1 RNA Sequencing

RNA sequencing (RNAseq) can be used to rapidly identify and quantify rare and common transcripts across a broad range of samples, providing information regarding gene expression, gene fusions, non-coding RNAs, exons, etc. (98).

#### 3.1.1 Differential Gene Expression

Bioinformatic data analyses combined with *in vitro* and *in vivo* experimental results have revealed that *MRPL13* is highly expressed in non-small cell lung cancer tissues and cell lines and can promote cancer cell proliferation; the locus is, therefore, an independent tumor marker and candidate therapeutic target (51). Expression levels of *MRPS6*, *MRPS10*, *MRPS23*, and *MRPS31* are significantly elevated in breast cancer cells and tissues, and these findings have been corroborated by corresponding cell-based functional assay results (52, 53). In addition, *MRPS23* has been identified as a driver of proliferation in luminal breast cancer, as supported by a series of *in vitro* and *in vivo* studies (54, 55). The depletion of MRPS29 functions *via* the β-Catenin/Lgr5/Bcl-2 axis to induce drug resistance in gastric cancer cells (99). *MRPL54*, *EZH2*, *PPARGC1A*, and *EIF2AK4* were identified as hub genes in bioinformatics analysis of hepatocellular carcinoma (HCC). RNA sequence data extracted from TCGA, and the gene signatures based on these loci showed good predictive ability for HCC prognosis (57, 58). Using a similar data mining approach, 12 genes, including *MRPS23*, were identified as promising predictors with an important role in the pathogenesis of colorectal cancer (CRC) (56). *MRPS12* expression is significantly higher in ovarian cancer than in normal ovarian tissues and is positively correlated with the infiltration of macrophages and neutrophils (59). Another bioinformatics analysis using GEO data revealed seven differentially expressed genes related to osteosarcoma metastasis, including *MRPS7*, but further experimental studies are still needed to elucidate the biological functions and mechanisms of action of these genes (60).

#### 3.1.2 Gene Fusions

Structural chromosomal rearrangements may result in the exchange of coding or regulatory DNA sequences between genes, and such gene fusions are strong driver mutations in the formation of malignant tumors, providing fundamental insights into mechanisms underlying tumorigenesis (100).

Fusion genes and epigenetic regulators (microRNAs, long non-coding RNAs, etc.) constitute an important part of the genomic landscape of tumors, including roles in CRC occurrence and progression. A tumor-specific gene fusion named *MRPS31-SUGT1*, generated by an intrachromosomal translocation on chromosome 13 and whose sequence was 100% identical to that of the lncRNA *MRPS31P5*, was discovered. *MRPS31P5* may be an important gene expression regulator and is a potential biomarker for detecting early CRC events (63). Intimal sarcoma is a rare, histologically heterogeneous tumor that usually originates from the pulmonary artery. Using data from the American Association for Cancer Research Project Genomics Evidence Neoplasia Information Exchange (AACRP GENIE) database, Roszik characterized genetic alterations in 13 patients with intimal sarcoma and their clinical value and identified genomic rearrangement events, especially the fusion of *MRPS30-ARID2* and *PDE4DIP-NOTCH2 *(64).

#### 3.1.3 Non-Coding RNAs

It is estimated that more than 90% of mammalian genomes are transcribed as non-coding RNAs. Long non-coding RNAs (lncRNAs) are broadly defined as non-coding RNA molecules longer than 200 nucleotides and lack the ability to encode proteins due to the lack of open reading frames (101, 102). LncRNAs located in nuclei regulate the expression of downstream target genes through epigenetic modification (103). The aberrant expression of lncRNAs leads to the development of many diseases such as cancer (104, 105).

lncRNAs have diverse functions in cancer immune responses and the tumor microenvironment. To investigate the immune-related lncRNA (IRlncRNA) signature for predicting bladder cancer (BLCA) prognosis and immunotherapy response, Wu extracted BLCA data from TCGA. Ultimately, eight IRlncRNAs with prognostic significance, including *MRPL23-AS1*, were identified as immunotherapy-related biomarkers (65). A recent study of oral squamous cell carcinoma has revealed a novel *LINC00152-USF1/MRPL52* signaling axis that promotes tumor growth. The long intergenic non-protein-coding RNA 152 (*LINC00152*) was identified as an oncogenic lncRNA in multiple cancers, which physically interacts with upstream transcription factor 1 (*USF1*) and can bind to the promoter region of *MRPL52* and activate its transcription (67). In another study, a risk score system based on five lncRNAs, including *MRPL23-AS1*, was used to predict the survival of patients with HCC with cirrhosis (66). Moreover, an *in-vitro* experimental study combined with a luciferase reporter assay revealed that the lncRNA *TRIM52-AS1* sponged *miR-514a-5p* to facilitate HCC progression through increasing *MRPS18A* expression (68).

#### 3.1.4 CeRNAs

Competing endogenous RNAs (ceRNAs) are transcripts that regulate each other at the post-transcriptional level by competing for shared miRNAs. ceRNA networks, which link the function of protein-coding mRNAs and noncoding RNAs, contribute to the pathogenesis of cancers (106). Furthermore, host microbiota contributes to many diseases, including cancer. Therefore, Xiangzhou Tan constructed a ceRNA network using data from TCGA and GEO databases to identify the mechanisms underlying microbiota-mediated CRC development and progression, among which four gene signatures (*MRPL23-AS1*, *FRMD6-AS2*, and *LIFR-AS1*) were identified as independent prognostic factors for CRC (107).

### 3.2 DNA Sequencing

DNA sequencing (DNAseq) is used to detect nucleotide alterations, providing DNA-level information, such as insertions, deletions, single nucleotide polymorphisms (SNPs), as well as methylation *status *(108).

#### 3.2.1 Single Nucleotide Polymorphisms

SNPs are single bases in the genome with variation in a population. SNP-based DNA microarray technology can measure allele-specific copy numbers at many different SNP sites in the genome and can be used to analyze genome-wide cancer-related structural variation (49, 109). *H19* is a 3.0 kb highly conserved lncRNA present on human chromosome 11; *H19* polymorphisms are associated with increased susceptibility to a variety of cancers. A study of three *H19* polymorphisms in 213 patients with hepatoblastoma demonstrated that the *rs2839698* and *rs3024270* polymorphisms are associated with decreased *MRPL23* antisense RNA 1 (*MRPL23-AS1*) expression, whereas the *rs217727* polymorphism is associated with increased *MRPL23-AS1* expression (69). Many studies have evaluated the association between the *miR-608 rs4919510* polymorphism and CRC susceptibility. The G allele of *rs4919510* located in *miR-608* is associated with increased expression of *MRPL43* in colon tissues (70). Functional experiments have shown that the knockdown of *MRPL43* could inhibit growth and promote apoptosis in the CRC HCT-116 cell line. In addition, SNP loci in *MRPS30* are associated with breast cancer (110–112).

#### 3.2.2 Methylation

DNA methylation is a covalent modification in which methyl groups are added to the cytosine of CpG islands in the genome by *S*-adenosylmethionine. As an epigenetic modification, methylation can regulate gene expression without altering the DNA sequence (113). DNA microarray-based high-throughput methylation sequencing provides information on epigenetic changes in the genome. In a study of neuroblastoma with the largest coverage to date, methylation-specific PCR assays for 78 differentially methylated regions revealed associations between event-free survival and five unique regions, including *lnc-MRPL3-2*; several novel prognostic biomarkers were identified and independently validated (71).

### 3.3 WGCNA

Weighted Gene Co-Expression Network Analysis (WGCNA) is a bioinformatics approach to determine intergenic associations in microarray samples, involving network construction, module detection, calculation of topological properties, and data simulation; this approach is useful for screening disease biomarkers or potential therapeutic targets (114). Co-expression is a commonly used strategy to analyze correlations. In the correlation network of genes, each node represents a gene, and lines between nodes are used to represent the correlation of the two genes. Traditional unweighted co-expression network calculates the correlation coefficient between two genes directly through a linear correlation function and judge whether there is a correlation between these two genes based on a given threshold of the correlation coefficient (115, 116). In fact, the correlation of two genes is a value that fluctuates from 0 to 1, and the traditional method ignores the originally changing trend, resulting in the unweighted co-expression network losing much information. Therefore, the development team of WGCNA optimized the algorithm. Pearson correlations are taken and then weighted by raising their absolute value to a power (“β” in adjacency function) (117). This operation guarantees the invariance of the correlation relationship and reinforces the level of variation of the correlation coefficient. Through this analysis, co-expressed gene modules can be identified, and furthermore, modules can be correlated with phenotypic data to mine potential mark genes through metrics such as connectivity, module membership (MM), and gene significance (GS) (117).

Using Dai’s study as an example, WGCNA was used to identify central genes associated with the incidence and prognosis of KRAS-mutant lung adenocarcinoma (LUAD). A total of 2590 DEGs were found between 184 LUAD samples of different pathological stages and 59 normal lung tissue samples from TCGA database, and 10 gene modules were identified. Functional analysis of the key modules revealed enrichment of ribosome biogenesis related terms. Survival analysis revealed that the expression of 8 genes, including MRPL27, was positively correlated with poor survival in patients with KRAS-mutant LUAD (61). Tumor mutational burden (TMB) is associated with the efficacy of immunotherapy, but the prognostic role of TMB-related genes in HCC has not been clearly defined. A WGCNA identified *MRPL9* as a TMB-specific gene with good prognostic value. A cell-based functional study has shown that the knockdown of *MRPL9* could significantly inhibit cell proliferation and migration in HCC (40). Cholangiocarcinoma (CCA), a highly malignant tumor found in biliary epithelial cells, is the second most common primary tumor of the liver. A WGCNA based on mRNA sequencing data and clinical information for patients with CCA (from TCGA) revealed that *MRPS18A, CST1*, and *SCP2* are genes associated with clinical features, such as pathological stage, histological grade, and liver function, as well as overall survival (62).

### 3.4 Mitochondrial Ribosomes and Clinical Features

TCGA sample collection and processing involved multiple collaborating centers, including Tissue Source Sites (TSSs), Biospecimen Core Resource (BCR), and Data Coordinating Center (DCC) (http://cancergenome.nih.gov/abouttcga/overview). Biological samples (blood and tissue) are collected from eligible cancer patients, who have detailed clinical information. Potential biomarkers are screened by follow-up high-throughput sequencing and bioinformatics analyses related to clinical features, such as prognosis, lesion metastasis, and drug responsiveness.

#### 3.4.1 Prognosis

Hu et al. identified 92 differentially expressed genes from TCGA and developed a gene signature and prognostic risk model based on *MRPL47, NCBP2, MKRN3, AZGP1, IGF2BP2*, and *EZH2*, providing a basis for personalized immunotherapy for patients with head and neck squamous cell carcinoma (72). *MRPL11* is highly expressed in stage 4 neuroblastoma and is associated with a poor prognosis. Tigecycline, an FDA-approved broad-spectrum antibiotic, may be an indirect therapeutic strategy for neuroblastoma *via* the dysregulation of *MRPL11 (* 73, 74).

Another study has demonstrated that high *MRPL15* expression predicts a worse prognosis in non-small cell lung cancer; it revealed potential regulatory networks and demonstrated a negative correlation with immune infiltration (75). *MRPL42* is highly expressed in early-stage lung adenocarcinoma (LUAD) tissues and cell lines and is significantly associated with prognosis. The knockdown of *MRPL42* reduces cell proliferation, promotes cell cycle arrest at the G1/S phase, and attenuates the migration and invasion abilities of LUAD cells *in vitro (* 76).

Breast cancer accounts for the largest number of datasets in TCGA. Yin et al. integrated several bioinformatics tools and RNA *in situ* detection to identify and validate breast cancer-related hub genes and found that the expression of four genes, including *MRPL3*, were upregulated in tumor tissues and correlated with cancer progression; these genes could serve as diagnostic and prognostic biomarkers (77). As a poor prognostic factor in breast cancer, *MRPL13* expression is significantly higher in cancer tissues than in normal tissues (78–80). This gene can promote tumor cell proliferation, migration, and EMT *via* the *PI3K-Akt-mTOR* pathway and is, therefore, a candidate therapeutic target and prognostic marker (81). In breast cancer, a three-gene prognostic model based on *MRPL12, MRPL13*, and *POP1* has significant predictive value for survival. Moreover, the downregulation of endogenous *MRPL12, MRPL13*, or *POP1* expression could significantly inhibit the viability and migration of breast cancer cells *in vitro (* 82).

Gastric cancer (GC) is a digestive system disease with high morbidity and mortality; early clinical screening and diagnosis are difficult, and treatment efficacy at a later stage is unsatisfactory. Therefore, it is imperative to find new tumor markers and therapeutic targets for GC. Based on GC data in TCGA and Genotype Tissue Expression (GTEX), aberrantly expressed proteins between normal and cancer tissues were identified, and hub genes, such as *MRPS5*, were found to be associated with disease-specific survival (83). *MRPL35* expression is upregulated in GC and is associated with a poor prognosis. Cell functional studies have revealed that the knockdown of *MRPL35* inhibits cell proliferation in GC *via* related proteins, including PICK1, Bcl-xL, and AGR2 (84). High expression of *MRPS29* in GC is associated with a better prognosis, and the downregulation of *MRPS29* expression could increase resistance to chemotherapy by inhibiting apoptosis and promoting cell migration, consistent with the pro-apoptotic function of *MRPS29 (* 85). In another study on gastric adenocarcinoma, a model based on seven genes, such as *MRPS17*, was identified as a reliable prognostic biomarker (86).

HCC is one of the most prevalent neoplasms and the leading cause of cancer-related deaths worldwide. An mRNA expression-based stemness index revealed a three-gene signature, including *PTDSS2, MRPL9*, and *SOCS*, as a potential prognostic biomarker for HCC (87). Based on the differentially expressed genes between HBV-related HCC and control specimens, screened by univariate analyses, a signature model based on 11 genes, including *MRPS12*, was finally developed. This gene signature showed high sensitivity and accuracy for the prediction of 1-, 3-, and 5-year overall survival, disease-free survival, and progression-free interval, with predictive power superior to those of other clinical parameters (88).

Cholangiocarcinoma, a highly malignant tumor found in biliary epithelial cells, is the second most common primary tumor of the liver. A study of 36 patients with cholangiocarcinoma demonstrated that *MRPL27* mRNA levels are significantly upregulated in tumor tissues, and patients with high *MRPL27* expression had a poorer overall survival and disease-free survival than those of patients with low *MRPL27* expression (89).

Additionally, 19 genes, including *MRPL52*, have been identified from microarray gene expression profiling data for 78 patients with CRC. Further validation using patient cohorts from Australia (n = 185), the United States (n = 114), Denmark (n = 37), and Norway (n = 95) revealed that this 19-gene signature has significantly better predictive value for survival in CRC than the traditional Dukes classification (90). Higher expression of *MRPL35* in CRC cells and tissues is associated with shorter overall survival in CRC, and *in vitro* studies have shown that the downregulation of *MRPL35* expression leads to increased production of reactive oxygen species (91).

Ovarian cancer (OC) is a leading cause of death among gynecological cancers, and unique tissue and genetic heterogeneity are huge obstacles to their diagnosis and treatment. Xu et al. screened six MRPs (uL10m, uL15m, bL36m, mL39, bS16m, and mS31) associated with OC, among which *MRPL15* (uL15m) was highly expressed and amplified in OC and associated with a poor prognosis. A mechanistic analysis revealed that *MRPL15* (uL15m) plays a role in OC *via* various pathways, including cell cycle, DNA repair, and *mTOR 1* signaling (92).

Adrenocortical carcinoma (ACC) is an extremely rare disease, and the current prognostic markers have limitations in identifying patients with poor prognosis. In a previous study, 45 formalin-fixed paraffin-embedded (FFPE) tissues of adrenal tumors were analyzed by liquid chromatography-tandem mass spectrometry (LC-MS/MS) to screen differentially expressed proteins by machine learning algorithms. *MRPS23* was found to be significantly associated with survival in ACC, and this finding was validated using the TCGA database; thus, *MRPS23* could be considered as a potential prognostic marker (93).

#### 3.4.2 Metastasis

Xinyan Li identified differentially expressed mRNAs in breast tumors with and without metastasis by high-throughput RNA sequencing, revealing that the expression of *MRPL52* was upregulated in breast cancer and was significantly associated with aggressive clinicopathological features and a higher risk of metastasis (94). Sotgia combined four mitochondrial proteins (uL15m, *HSPD1*, *UQCRB*, and *COX17*) to generate a compact mitochondrial gene signature that successfully predicts distant metastasis in breast cancer, suggesting that mitochondrial biogenesis should be considered a novel therapeutic target to overcome tumor recurrence, distant metastasis, and treatment failure in breast cancer (95). Studies targeting human OC cell lines SKOV3 and SKOV3.ip1 with different metastatic potential revealed differential expression in 11 genes, such as *MRPL38*, suggesting that high *MRPL38* expression is associated with invasion and metastasis (96). Consistent with these findings, evaluation of data from the Human Protein Atlas revealed that the protein abundance of *MRPL38* (mL38) is highly elevated in most tumor types (1).

#### 3.4.3 Drug Responsiveness

Cisplatin-based neoadjuvant chemotherapy (NAC) prior to radical cystectomy is recommended for patients with muscle invasive bladder cancer (MIBC). However, clinically approved biomarkers for predicting the response to NAC are lacking. A study involving 30 MIBC cases reported a new signature gene set, including *MRPL4*, and was able to select NAC responders with 100% prediction accuracy; this gene set could serve as a promising biomarker for predicting chemo-responsiveness in patients with MIBC (97).

## Concluding Remarks

Mitochondrial ribosomes are involved in biological activities *via* the regulation of energy metabolism and apoptosis. Next-generation sequencing and novel bioinformatics analyses have revealed that mitochondrial ribosomal genes are closely related to malignancy. Given the potential of mitochondrial ribosomal genes as cancer biomarkers, the detection of abnormally expressed mitochondrial ribosomal coding genes and non-coding RNAs is expected to facilitate the early diagnosis of malignant tumors. In the future, therapies that specifically target mitochondrial ribosomes may improve the prognosis of patients with cancers.

## Author Contributions

SB, XW, and ML: induction, analysis, writing, and financial support. ZG, DZ, and DS: literature search and curation. LL: guidance and modifications. All authors contributed to the article and approved the submitted version.

## Funding

This work was supported by a grant from the National Natural Science Foundation of China (No. 81773523).

## Conflict of Interest

The authors declare that the research was conducted in the absence of any commercial or financial relationships that could be construed as a potential conflict of interest.

## Publisher’s Note

All claims expressed in this article are solely those of the authors and do not necessarily represent those of their affiliated organizations, or those of the publisher, the editors and the reviewers. Any product that may be evaluated in this article, or claim that may be made by its manufacturer, is not guaranteed or endorsed by the publisher.

## Abbreviations

mtDNA, mitochondrial DNA; rRNA, ribosomal RNA; tRNA, transfer RNA; LSU, Large Subunit; SSU, Small Subunit; MRPs, Mitochondrial Ribosomal Proteins; OXPHOS, Oxidative Phosphorylation; DAP3, Death-Associated Protein 3; GEO, Gene Expression Omnibus; TCGA, The Cancer Genome Atlas; WGCNA, Weighted Gene Co-Expression Network Analysis; RNAseq, RNA sequencing; ceRNAs, competing endogenous RNAs; DNAseq, DNA sequencing; HCC, Hepatocellular Carcinoma; CRC, Colorectal Cancer; OC, Ovarian Cancer; BLCA, Bladder Cancer; SNP, Single Nucleotide Polymorphisms.
